# Case Report: A case report of unstable Hangman fracture in a eighty year old male

**DOI:** 10.12688/f1000research.6799.2

**Published:** 2015-12-17

**Authors:** Sunil Munakomi, Binod Bhattarai

**Affiliations:** 1 Department of Neurosurgery, College of Medical Sciences, Bharatpur, Nepal

**Keywords:** hangman’s fracture, unstable, management

## Abstract

Herein we discuss a rare variant of hangman’s fracture in an eighty year old male with good Karnofsky performance score. We performed X-ray and magnetic resonance imaging (MRI) of the cervical spine to confirm the diagnosis. The patient was placed on a gentle cervical traction which showed good reduction. Despite being on a resource limited setup,  we performed posterior occipitocervical fusion with bone graft fusion followed by early mobilization. A postoperative scan showed good reduction and purchase of the screws. This case highlights the importance of choosing the correct therapeutic attitude for the management of the geriatric population especially in those who do not have any significant co-morbid conditions.

## Introduction

Rigid immobilization alone is sufficient for most cases of hangman’s fracture (defined as traumatic spondylolisthesis of C2) classified as Effendi type I and some of type II. Effendi type III fractures are very rare and invariably have neurological deficits because of impingement due to the facet dislocation on the spinal cord posteriorly ^1^. Fracture instability is the presence of complete disruption of the annular and/or posterior ligament with forward and/or rotatory vertebral body slip of axis ^2^. Surgical stabilization and rigid immobilization together is recommended in such cases, such as Levine-Edwards type IIa and III fractures. Here we discuss the management of an unstable type III hangman’s fracture in an aged patient without any neurological deficits. Most doctors choose traction and prolonged immobilization in a halo vest due to associated medical comorbidities and the anesthetic risks involved in this group ^3, 4^. However there is a high risk of nonunion, instability, persistent pain and a need for a prolonged period of halo immobilization ^5^. Since our patient had a good Karnofsky performance score ^6^, we opted for only posterior fusion so as to minimize the anesthetic risk involved with both anterior and posterior approaches. However, we chose a long segment occipitocervical screw and graft fusion so as to aid the healing process in the aged bone.

## Case report

**Figure 1.**
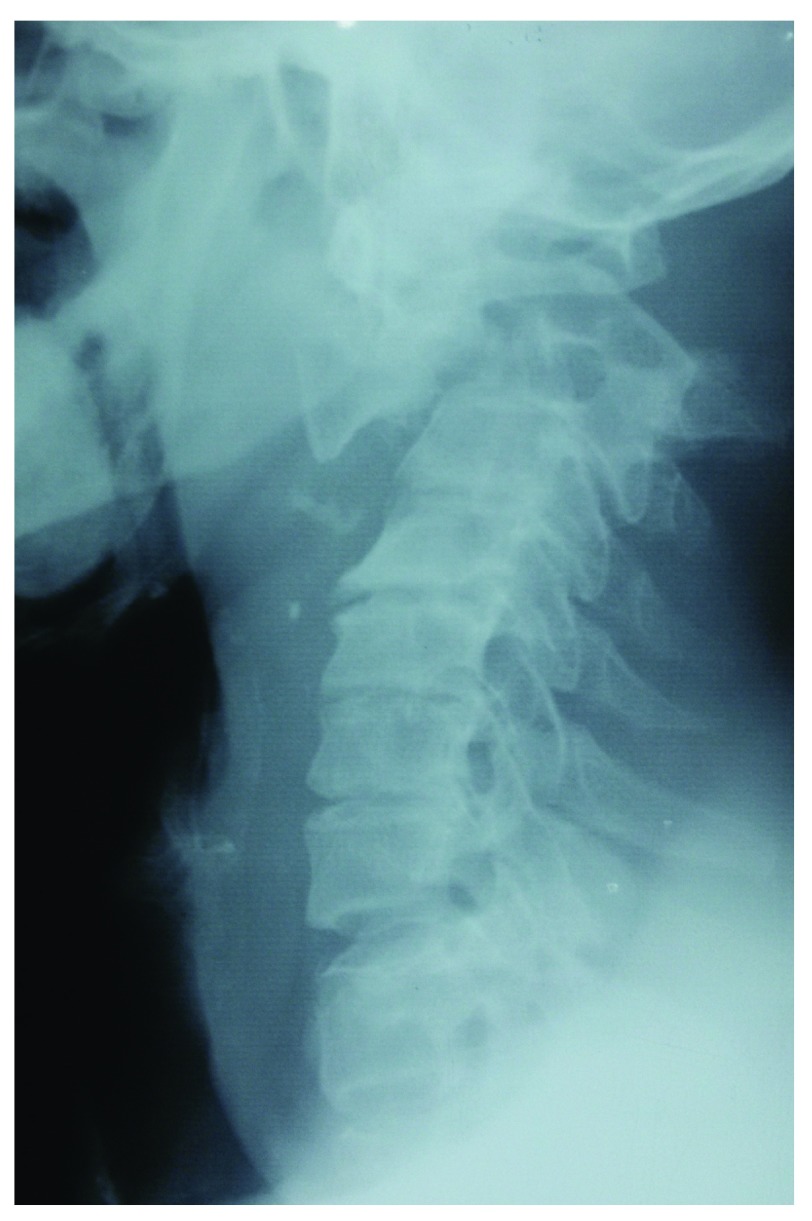
X-ray of the cervical spine showing Hangman’s fracture with significant translation and angulation.

**Figure 2.**
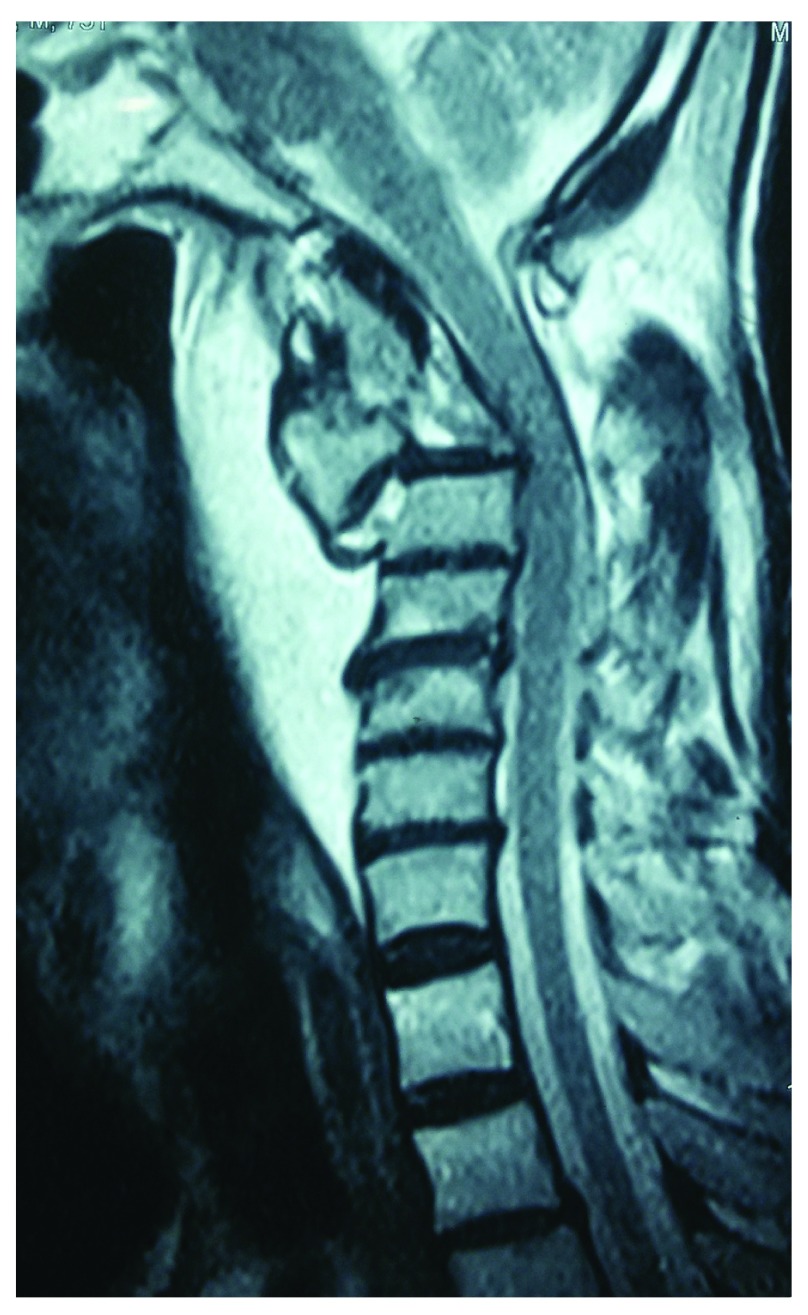
MRI (t2 sequence) of the cervical spine revealing presence of pinching effect but no signal changes in the cord.

An 80 year old man from the Tarai region of Nepal was brought to emergency with the chief complaint of falling from a swing after being pushed by his grandson 2 days prior. He complained of pain at the nape of his neck. The patient was neurologically intact. He was placed in a cervical collar and an urgent X-ray of the cervical spine revealed presence of spondylolysthesis of the axis with significant translation and angulation ( Figure 1). Magnetic resonance imaging (MRI) of the cervical spine revealed a type III hangman’s fracture with presence of pinching effect on the cord without any significant signal changes ( Figure 2).

**Figure 3.**
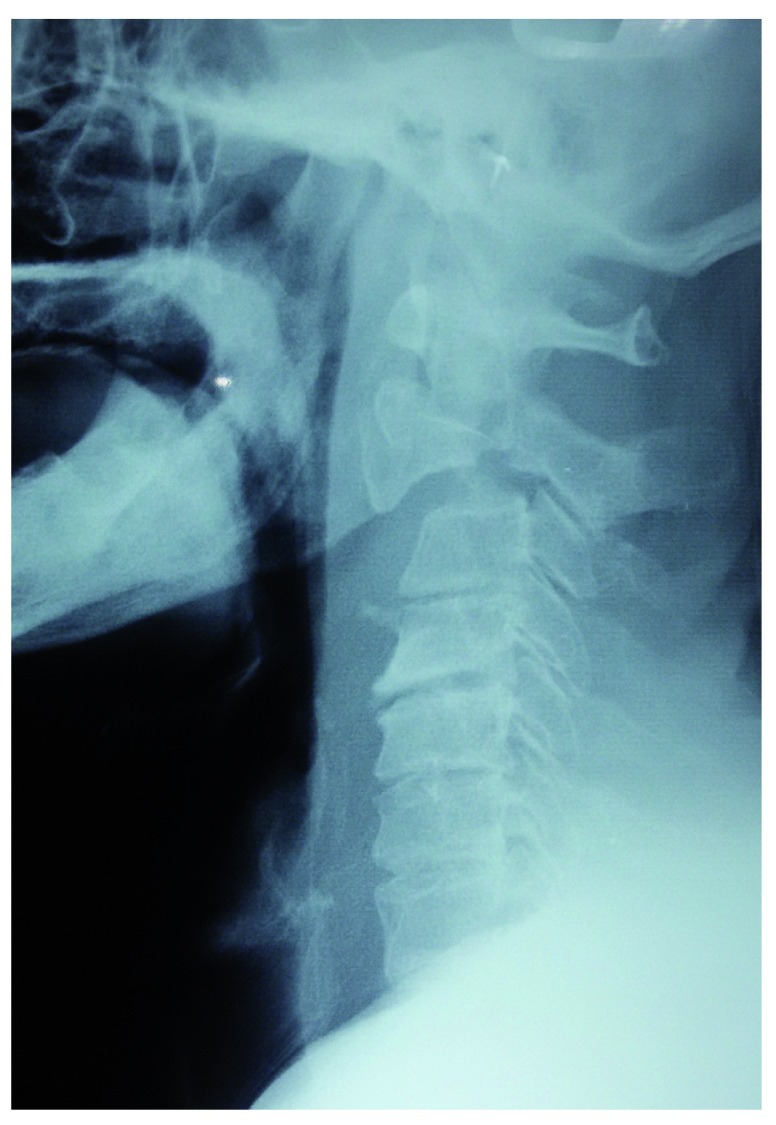
X-ray spine after traction showing realignment of the posterior and the spino-laminar lines.

**Figure 4.**
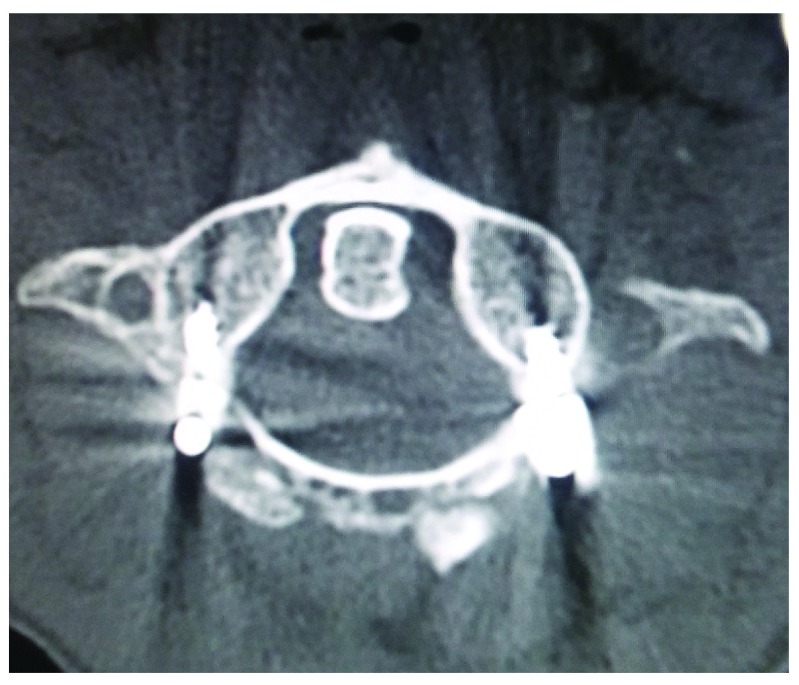
CT spine showing projection of screws through lateral mass of C1.

**Figure 5.**
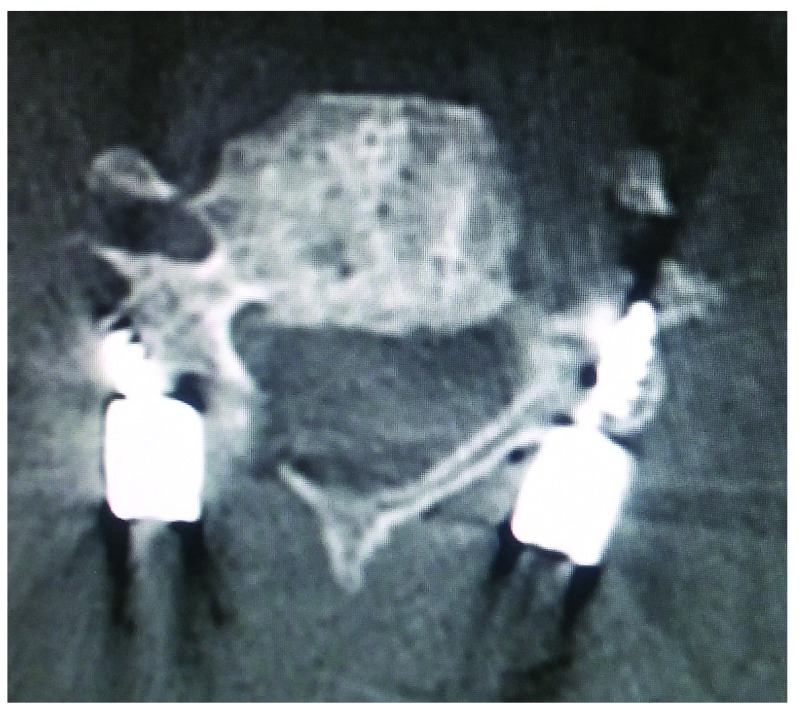
CT spine showing projection of screw through lateral mass of C3.

**Figure 6.**
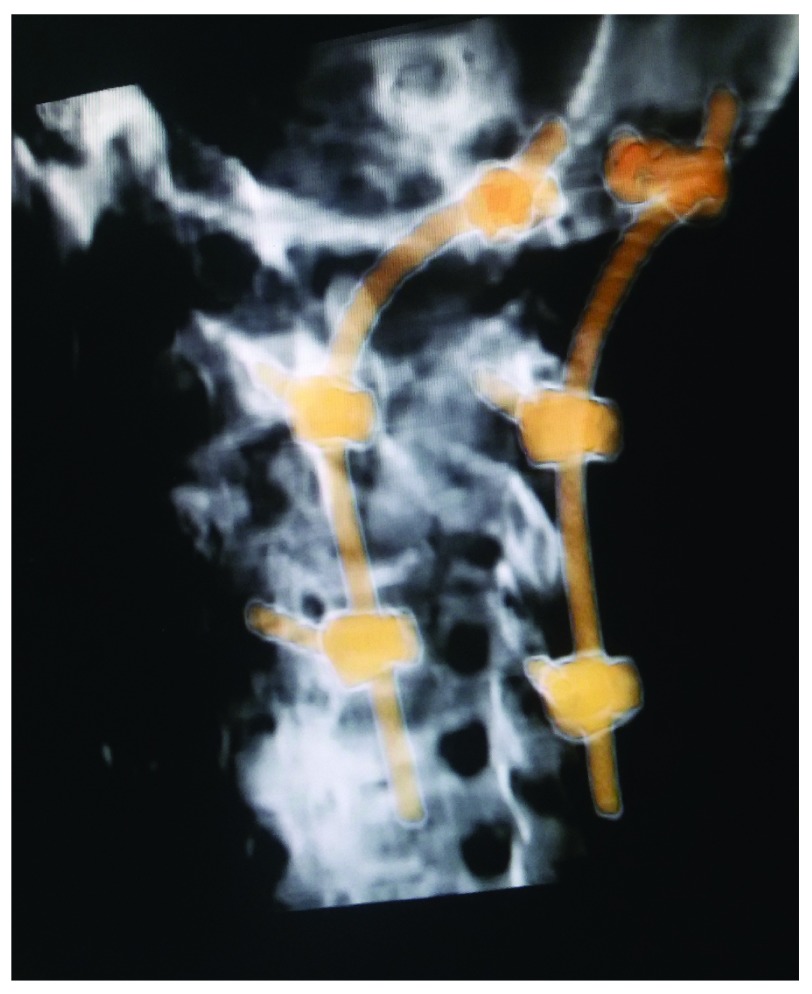
CT spine reconstruction showing projection and final alignment of the construct.

**Figure 7.**
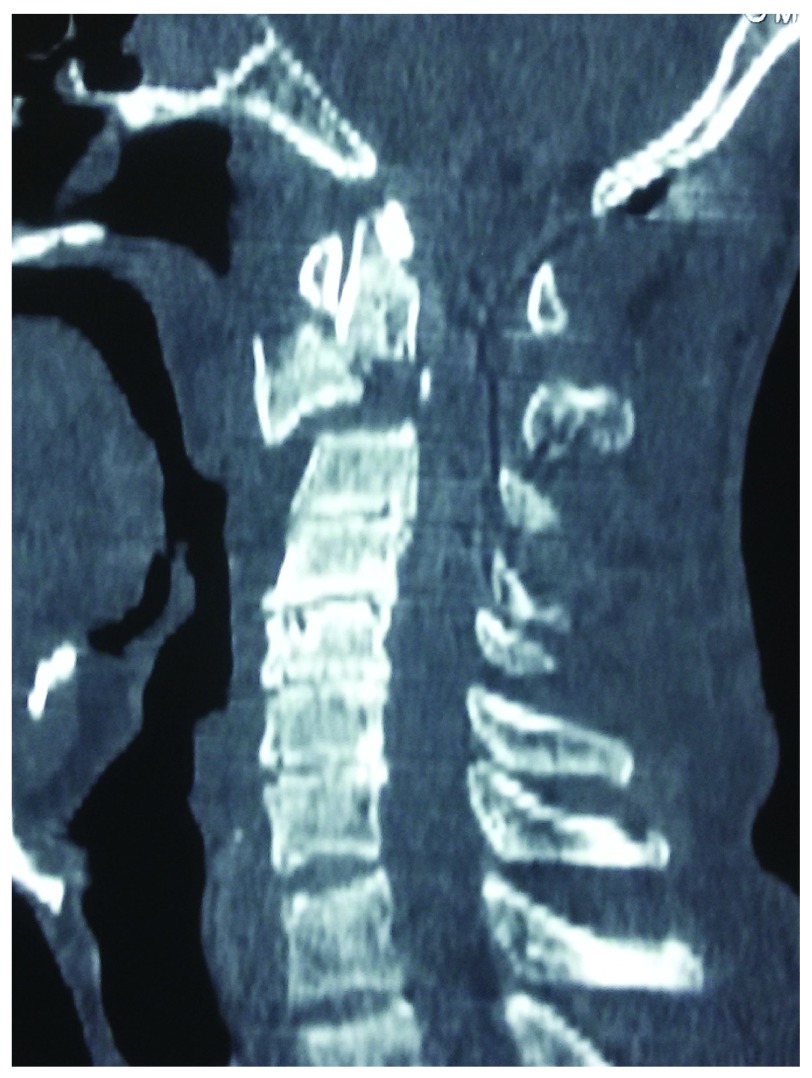
CT spine showing good reduction of the posterior and the spino-laminar lines and normal canal.

The patient was an ex-army serviceman and was in good health with good Karnofsky performance score ^6^. There was no significant past medical or surgical illnesses. He had a habit of smoking marijuana previously. However, routine screening echocardiography revealed a cardiac ejection fraction of only 33%. Because the fracture was an unstable type III variant, the decision of surgical fixation was taken. The best option in such a situation would have been anterior cervical disectomy and Casper plate fixation thereby avoiding prolonged prone positioning. However such armamentarium for the procedure was not available with us. After explaining the disease condition, treatment options available and the risks involved, the patient was placed on minimal cervical traction so as to avoid the risk of iatrogenic hanging. We looked for the level of realignment that was possible with the guarded traction. Stringent care was taken to observe for features of over distraction. Repeat imaging showed good realignment and normal canal diameter. Therefore we decided to go for occipito-cervical fusion so as to minimize the anesthetic risk imposed to the patient from both anterior and posterior approaches. A DEXA scan for assessing bone density would have been justified prior to occipito-cervical fusion since such procedure would further lead to severe motion restriction in such an elderly spine. However such facility was not available to us. Intra-operatively there was fracture of the pars and the lamina of C2. Since there was no atlantoaxial dislocation, we opted for occipital and C1 and C3 lateral mass fixation. There is evidence of good results with short fixation of C1 and C3 only, but keeping in mind the risk of osteoporosis in this case, we wanted further anchorage from occipital fusion as well. Since there was good posterior realignment of the spinal lines after traction ( Figure 3) and intra-operatively, we choose the posterior approach only to minimize the added risk of the anterior approach. Lateral mass screws were placed in C1 and C3 ( Figure 4 and Figure 5). Bone graft harvested from iliac bone was placed in the C1 and C2 inter-space to further enhance the fusion process. The patient was started on dexamethasone (8 mg intravenously and then rapidly tapered off in the following 2 days). The patient was safely extubated. Neurological examination was normal. The patient was in complete bed rest for a week and then mobilized with support. A CT spine check after one week revealed good screw purchase ( Figure 6) and good reduction of fracture segment ( Figure 7). The patient was restricted to light weight bearing and was advised to keep the cervical collar for at least 6 weeks. The patient was started on calcium supplementation (tablet calcium 500 mg orally every 12 hours. The patient followed up in the outpatient department after 1.5 months walking on his own without any deficits.

## Discussion

“Hangman's fracture”, a traumatic spondylolisthesis of C2, first coined by Schneider *et al.* in 1965 ^7^ results from hyperextension of the upper cervical spine. There is fracture of the lateral mass and the pedicle of the axis with simultaneous disruption of the anterior longitudinal ligament allowing C2-C3 listhesis. Traumatic hangman’s fracture, in contrast to the judicial hangman's fracture, is caused from extension and compression of the upper cervical spine with rare cord injury ^8^.

The most widely used classification for hangman's fractures was firstly described by Effendi *et al.* ^9^ and later modified by Levine *et al.* ^10, 11^. Anterior approaches include anterior cervical disectomy and graft fusion ^12^; posterior approaches include lateral mass, pedicle or transarticular screw placement ^13^.

Anterior discectomy and screw plate fixation is an effective, but not very popular technique due to difficulty in exposing the C2-C3 region ^14^ and the elimination of C2-C3 rotation ^15^. Direct screw fixation of C2 pars adds to the risk of injury to the vertebral artery ^15^ and also there is the need for complete manual reduction of the fracture intra-operatively ^15, 16^.

Fusion of lateral masses of C1 and C3 for hangman's fractures minimizes risk of vertebral artery injury and displacement of fractured segments into the canal. The efficacy of this approach has been validated in a biomechanical study by Chittiboina *et al.* ^17^

This study hereby highlights the importance of the treatment algorithm chosen for the management of unstable hangman’s fracture in geriatric patients. Patients with good Karnofsky performance score would benefit from long segment posterior fusion, rather than both anterior and posterior approaches which might increase the intra-operative risk. Managing such patients with a prolonged period of immobilization in a halo imposes a higher risk of nonunion.

## Conclusion

Age alone should not determine a doctor’s approach to the treatment of geriatric patients. By taking only age into account when deciding on treatment, we risk compromising effective management in elderly patients. Karnofsky performance scale ^6^ is one reliable marker that helps in making such treatment decisions. So despite being on a resource limited setup, we can tailor ourselves into adopting other viable options.

## Consent

Both written and verbal informed consent for publication of images and clinical data related to this case was sought and obtained from the son of the patient.
