# Supplementary material for: Case Report: A case report of unstable Hangman fracture in a eighty year old male
Source: F1000Res. 2015 Dec 17;4:337. Originally published 2015 Jul 23. [Version 2] doi: 10.12688/f1000research.6799.2 (PMC4722693; doi:10.12688/f1000research.6799.2)
Supplement: Peer review history

# Peer review: Referee response for version 2

**DOI:** 10.5256/f1000research.7957.r11993

**Published:** 2016-01-20

**Authors:** Virendra Deo Sinha

**Affiliations:** 1 Department of Neurosurgery, S.M.S. Medical College, Jaipur, Rajasthan, India

The authors have successfully amended the article to the queries and reservations which we had made earlier

I have read this submission. I believe that I have an appropriate level of expertise to confirm that it is of an acceptable scientific standard.

# Peer review: Referee response for version 2

**DOI:** 10.5256/f1000research.7957.r11617

**Published:** 2015-12-30

**Authors:** George Wittenberg

**Affiliations:** 1 Department of Neurology, University of Maryland School of Medicine, Baltimore, MD, USA

The authors have been very responsive to the comments. My statements before stand about the overall value of the paper. There is some idiosyncratic use of English, including the concluding statement about being able to "tailor ourselves into adopting other viable options." One could rewrite it as: 

"So despite having limited resources, we can explore viable options."

I have read this submission. I believe that I have an appropriate level of expertise to confirm that it is of an acceptable scientific standard.

# Peer review: Referee response for version 1

**DOI:** 10.5256/f1000research.7308.r9611

**Published:** 2015-12-03

**Authors:** George Wittenberg

**Affiliations:** 1 Department of Neurology, University of Maryland School of Medicine, Baltimore, MD, USA

I think this is a well-written and interesting case report. As a single case report, it cannot be used to guide treatment. The outcome could have been different even though the authors suggest a very reasonable approach to the situation. I would agree with Dr. Ratan's comments, although I may be less of an expert on the surgical management of hangman's fracture. I think there would be a small benefit to medical knowledge to indexing this paper, as the management of this condition would likely be guided by both exam, radiological findings, and patient medical condition.

I have read this submission. I believe that I have an appropriate level of expertise to confirm that it is of an acceptable scientific standard, however I have significant reservations, as outlined above.

# Peer review: Referee response for version 1

**DOI:** 10.5256/f1000research.7308.r11119

**Published:** 2015-11-06

**Authors:** Virendra Deo Sinha, Amit Chakrabarty

**Affiliations:** 1 Department of Neurosurgery, S.M.S. Medical College, Jaipur, Rajasthan, India; 2 Neurosurgery Department, Eternal Hospital, Jaipur, Rajasthan, India

We have read with interest the case report "A case report of unstable Hangman fracture in a eighty year old male" by Munakomi *et al *.The authors need to be congratulated for highlighting their view in managing unstable Hangman fracture in the geriatric age group.

The patient in your report was put on traction with six kilogram loading. It is an accepted fact that patients with type 3 Hangman fracture should not be subjected to traction due to risk of “iatrogenic hanging’’.

Secondly, the radiology images published do not point towards severe osteoporosis. A DEXA scan could have been more informative as the decision to do occipito-cervical fusion in Hangman fracture exposes the patient to severe motion restriction in an already spondylotic spine.

Finally, I presume C2 -C3 anterior cervical discectomy and fixation with casper plates would have been preferable than to subjecting the patient with an ejection fraction of 33% to surgery in prone position.

We have read this submission. We believe that we have an appropriate level of expertise to confirm that it is of an acceptable scientific standard, however we have significant reservations, as outlined above.

# Peer review: Referee response for version 1

**DOI:** 10.5256/f1000research.7308.r9613

**Published:** 2015-08-11

**Authors:** Rajiv Ratan

**Affiliations:** 1 Burke Medical Research Institute, Weil Medical College of Cornell, White Plains, NY, USA

This report describes a case of a Hangman's fracture without associated neurological symptoms. 

There are several problems with this report that limit its usefulness and message, 

First, a Hangman's fracture, which is defined as fractures of both pedicles or pars interarticularis of the C2 vertebra (axis), is not defined in the abstract. There is a classification protocol that has been developed by Levine and Edwards that is based on etiology and guides treatment, this is also not mentioned. 

Second, neurological impairment is only seen in 25% of cases, so the absence of neurological symptoms is the rule not the exception as implied by this abstract. 

I am not convinced that this case report adds significantly to our understanding of how to diagnose and treat Hangman's fracture.

I have read this submission. I believe that I have an appropriate level of expertise to state that I do not consider it to be of an acceptable scientific standard, for reasons outlined above.

# Response

**Published:** 2015-08-11

**Authors:** Sunil Munakomi

Thank you for the report. We totally agree with your comments, but the purpose of our paper was to highlight the clinical importance of choosing the correct surgical management, barring the age factor, which do significantly play a major role in decision making in developing countries like ours. So, we wanted to highlight the importance of neurological status and the Karnofsky score in making such decisions in geriatric patients.
